# Efficacy of Oral Metronidazole with Vaginal Clindamycin or Vaginal Probiotic for Bacterial Vaginosis: Randomised Placebo-Controlled Double-Blind Trial

**DOI:** 10.1371/journal.pone.0034540

**Published:** 2012-04-03

**Authors:** Catriona S. Bradshaw, Marie Pirotta, Deborah De Guingand, Jane S. Hocking, Anna N. Morton, Suzanne M. Garland, Glenda Fehler, Andrea Morrow, Sandra Walker, Lenka A. Vodstrcil, Christopher K. Fairley

**Affiliations:** 1 Melbourne School of Population Health, University of Melbourne, Melbourne, Australia; 2 Melbourne Sexual Health Centre, The Alfred Hospital, Melbourne, Australia; 3 Department of Epidemiology and Preventive Medicine, Monash University, Melbourne, Australia; 4 Department of General Practice, University of Melbourne, Melbourne, Australia; 5 Centre for Women's Health, Gender and Society, University of Melbourne, Melbourne, Australia; 6 Department of Microbiology and Infectious Diseases, The Royal Women's Hospital, Melbourne, Australia; 7 Department of Obstetrics and Gynaecology, University of Melbourne, Melbourne, Australia; 8 Department of Microbiology, The Royal Children's Hospital, Melbourne, Australia; Assiut University Hospital, Egypt

## Abstract

**Background:**

To determine if oral metronidazole (MTZ-400mg bid) with 2% vaginal clindamycin-cream (Clind) or a *Lactobacillus acidophilus* vaginal-probiotic containing oestriol (Prob) reduces 6-month bacterial vaginosis (BV) recurrence.

**Methods:**

Double-blind placebo-controlled parallel-group single-site study with balanced randomization (1∶1∶1) conducted at Melbourne Sexual Health Centre, Australia. Participants with symptomatic BV [Nugent Score (NS) = 7–10 or ≥3 Amsel's criteria and NS = 4–10], were randomly allocated to MTZ-Clind, MTZ-Prob or MTZ-Placebo and assessed at 1,2,3 and 6 months. MTZ and Clind were administered for 7 days and Prob and Placebo for 12 days. Primary outcome was BV recurrence (NS of 7–10) on self-collected vaginal-swabs over 6-months. Cumulative BV recurrence rates were compared between groups by Chi-squared statistics. Kaplan-Meier, log rank and Cox regression analyses were used to compare time until and risk of BV recurrence between groups.

**Results:**

450 18–50 year old females were randomized and 408 (91%), equally distributed between groups, provided ≥1 NS post-randomization and were included in analyses; 42 (9%) participants with no post-randomization data were excluded. Six-month retention rates were 78% (n = 351). One-month BV recurrence (NS 7–10) rates were 3.6% (5/140), 6.8% (9/133) and 9.6% (13/135) in the MTZ-Clind, MTZ-Prob and MTZ-Placebo groups respectively, p = 0.13. Hazard ratios (HR) for BV recurrence at one-month, adjusted for adherence to vaginal therapy, were 0.43 (95%CI 0.15–1.22) and 0.75 (95% CI 0.32–1.76) in the MTZ-Clind and MTZ-Prob groups compared to MTZ-Plac respectively. Cumulative 6-month BV recurrence was 28.2%; (95%CI 24.0–32.7%) with no difference between groups, p = 0.82; HRs for 6-month BV recurrence for MTZ-Clind and MTZ-Prob compared to MTZ-Plac, adjusted for adherence to vaginal therapy were 1.09(95% CI = 0.70–1.70) and 1.03(95% CI = 0.65–1.63), respectively. No serious adverse events occurred.

**Conclusion:**

Combining the recommended first line therapies of oral metronidazole and vaginal clindamycin, or oral metronidazole with an extended-course of a commercially available vaginal-*L.acidophilus* probiotic, does not reduce BV recurrence.

**Trial Registration:**

ANZCTR.org.au ACTRN12607000350426

## Introduction

Bacterial vaginosis (BV) is the commonest cause of abnormal vaginal discharge in women of reproductive age, with a prevalence of 29% in 17–49 year old North American women [1]. First line therapies include a week of oral metronidazole or vaginal clindamycin cream, or 5 days of vaginal metronidazole gel, and all have similar short-term efficacy [2], [3]; however, long term BV-recurrence following these therapies is common, with rates as high as 58% within 12 months of oral metronidazole [4].

Unacceptably high BV-recurrence rates after monotherapy, and differing antibiotic susceptibilities of BV-associated bacteria, led researchers to investigate whether combining oral metronidazole and vaginal clindamycin was more effective than metronidazole monotherapy. An alternative or complementary approach to the use of antibiotics is the use of lactobacillus-probiotics to help restore the healthy vaginal ecosytem. A recent Cochrane review highlighted the need for large randomized placebo-controlled trials with standardized outcomes in this area [5]. We chose a commercially available vaginal-probiotic containing *Lactobacillus acidophilus* KS400 and 0.03 mg of oestriol to evaluate with metronidazole. The probiotic had demonstrated efficacy against BV in two studies, and shown normalization of vaginal flora with an increase in lactobacillus-scores in women with a BV when used after antimicrobial therapy [6], [7]. The inclusion of oestriol in the product is supported by the protective effect against BV observed in women using combined hormonal contraceptives such as the oral contraceptive pill and Nuvaring® in observational studies [4], [8], [9], [10], [11].

The aim of our study was to determine whether the combination of oral metronidazole with either 2% vaginal clindamycin cream or a commercially-available vaginal-probiotic reduced rates of BV-recurrence over 6 months compared to oral metronidazole with a vaginal-placebo.

## Methods

The protocol for this trial and supporting CONSORT checklist are available as supporting information; see Checklist S1 and Protocol S1.

### Trial Design

This was a double-blind placebo-controlled parallel-group study with balanced randomization (3 arms 1∶1∶1) conducted in accordance with the original protocol in a single site in Melbourne, Australia.

### Participants

Recruitment was conducted from December 2007 to May 2010 at the Melbourne Sexual Health Centre (MSHC). Eligible participants were 18–50 year old females with symptomatic BV, defined as abnormal vaginal discharge or odour with a Nugent Score (NS) of 7–10 or ≥3 Amsel's criteria and a NS of 4–10. Women were ineligible if they were: HIV positive, pregnant, breastfeeding, attempting to conceive, not fluent in English, without an Australian-postal address, and not able to abstain from vaginal sex during vaginal therapy if they were reliant on 100% condom use for STI protection/contraception. The mineral oil in clindamycin cream has been reported to affect condom integrity (Pfizer, Australia).

Symptomatic BV-positive women were examined by a clinician who recorded clinical signs and collected a high-vaginal swab for a vaginal wet-preparation and Gram-stained smear. The smear was scored by Nugent criteria in an onsite laboratory, and Amsel's criteria were recorded. Women were screened for *Chlamydia trachomatis* by Strand-displacement amplification (BD ProbeTec ET CT-Amplified DNA Assay, Becton-Dickinson & Co, MD,USA), *Neisseria gonorrhoeae* by culture of endocervical swabs (modified Thayer-Martin medium), *Trichomonas vaginalis* by wet preparation and culture of vaginal swabs (modified Diamonds medium) and *Candida* spp. by microscopy and culture. Women were referred to the research nurse, who obtained written informed consent from eligible participants. Participants self-completed a questionnaire on demographic, clinical and behavioural characteristics during the recruitment interview.

### Interventions

Participants received 400 mg twice daily of oral metronidazole for 7 days, and were randomly assigned to a vaginal intervention (placebo, clindamycin, or probiotic). Vaginal 2% clindamycin cream was in a plain white tube and prescribed as one applicator vaginally for 7 nights. The probiotic and placebo were prescribed as a single vaginal pessary for 12 nights in keeping with the manufacturer Medinova's (Switzerland) recommendations and freely-donated. The probiotic contained at least 10^7^ colony forming units (CFU) of live *L.acidophilus* KS400, 0.03 mg oestriol and excipients. The vaginal-placebo preparation was identical in appearance to the probiotic, and contained only excipients.

Vaginal preparations were packed according to the allocation sequence by a nurse with no other involvement in the study in sequentially-numbered opaque cardboard boxes. Samples were taken from the probiotic and placebo batches that were given to participants, and were cultured at two time points during the study. Under the same culture conditions, we confirmed no growth of bacteria in the placebo, and viability of ≥10^7^ CFU of live *L.acidophilus* but no growth of other bacteria in the probiotic.

### Outcomes

The primary outcome measure was recurrence of BV (NS 7–10) within six months, assessed at 1, 2, 3 and 6 months following the intervention by participant self-collected vaginal-smears. The use of self-collected samples for the diagnosis of BV has been shown to be comparable to clinician-collected samples [12], [13]. The secondary outcome measure was recurrence of abnormal vaginal-flora (NS 4–10). One of three blinded microscopists experienced in Nugent's methodology scored vaginal smears. Difficult to interpret slides were independently reread by the second then third microscopist until consensus was reached. In an audit during the trial, the three microscopists achieved ≥92% concordance in Nugent scoring a sample of 40 vaginal Gram-stained smears representing all Nugent categories. Participants were informed when they reached the primary endpoint (NS 7–10) and offered re-treatment with either metronidazole or clindamycin.

### Sample Size

We estimated a 6 month BV recurrence rate of 50% in the oral metronidazole/vaginal-placebo arm from previous work [4]. To detect a 20% reduction in BV recurrence with either active vaginal therapy compared to placebo, with a two-sided 5% significance level and 90% power, a sample size of 123 was required for each group. Assuming 20% loss to follow-up, 150 women were recruited to each treatment group.

### Randomization, Allocation concealment, Implementation and Blinding

Participants were randomly assigned to one of three study arms in blocks of 15 using a computer-generated sequence. The sequence was produced by a statistician with no clinical input into the trial and securely held by the statistician and nurse packing the vaginal therapies in sealed boxes. At enrolment a research nurse, with no access to the randomization schedule, gave each woman the next sequentially-numbered sealed box according to the random number sequence. Specific instructions for use of vaginal products were inside the sealed box, which participants were instructed to open at home. They were told their vaginal treatment may be an antibiotic, placebo or probiotic, that it may be a cream or pessary, and that the duration of use may be 7 or 12 days, but given no further product details. The participants, the research nurse co-ordinating enrolment and retention of study participants, investigators and microscopists were unaware of the participants' group allocation. The effectiveness of blinding was tested at completion of the trial when participants were asked to indicate in their questionnaire which vaginal treatment they thought they had received.

A data safety and monitoring board was not deemed necessary. Trial products had been licensed for use in Europe and/or North America, there is extensive community experience with oral metronidazole and vaginal clindamycin, and significant adverse events were considered unlikely. Participants were aware that they could attend MSHC or call a free investigator-staffed number for clinical queries seven days a week.

### Follow-up and retention

At each follow-up, participants were posted a kit containing a questionnaire, self-swabbing instructions, an encased-Dacron swab and glass slide to self-smear. Participants who developed symptoms between scheduled follow-ups were encouraged to call the free number staffed by investigators and to have interim samples collected, or to attend our clinical service. To optimize retention, participants were contacted by email or telephone upon kit postage and on three consecutive weekly occasions if the kit was not returned. Outcome data were deemed missing if not returned by halfway through the follow-up interval, or at 6 months if >210 days after enrolment, to allow sufficient time for return of final specimens/questionnaires. Participants who did not return specimens after these time points were deemed lost to follow-up and censored at their last returned-specimen.

### Statistical methods and ethics approval

Data were entered and stored in Microsoft Access and analysed using SPSS 17.0 (SPSS Inc.,Chicago,IL,USA) and STATA 9.0 (StataCorp,2005).. Participants were excluded if no post-randomization data was available, and individuals providing post-randomization data who were subsequently lost to follow-up, were analysed according to the last Nugent score available. Cumulative recurrence of both BV and abnormal flora was determined for each treatment group and the overall study population. Proportions were compared using Chi-square and Fisher's Exact tests where appropriate, and 95% confidence intervals (CIs) were calculated. All statistical tests were two-sided and a level of p<0.05 was considered significant. Kaplan-Meier methods were used to generate survival curves for time until recurrence of BV and abnormal vaginal-flora, and log rank analysis was used to compare survival curves between groups. Cox regression analysis was used to generate hazard ratios for risk of BV-recurrence. Multivariate analysis was performed to account for any differences in baseline characteristics between treatment groups, and for factors known to be clinically-associated with BV-recurrence. Longitudinal factors associated with BV-recurrence among trial participants will be explored in a subsequent paper. Data were censored when participants either had BV-recurrence, reached study endpoint at 210 days, or were deemed lost to follow-up, as previously described. This trial received ethics approval from the Human Research and Ethics Committees of the Alfred Hospital and Monash University, Melbourne, Australia, and written informed consent was obtained from all participants. The trial was registered at the Australian New Zealand Clinical Trials Registry [ACTRN12607000350426].

## Results

### Participant flow and recruitment baseline data

**Figure 1:**
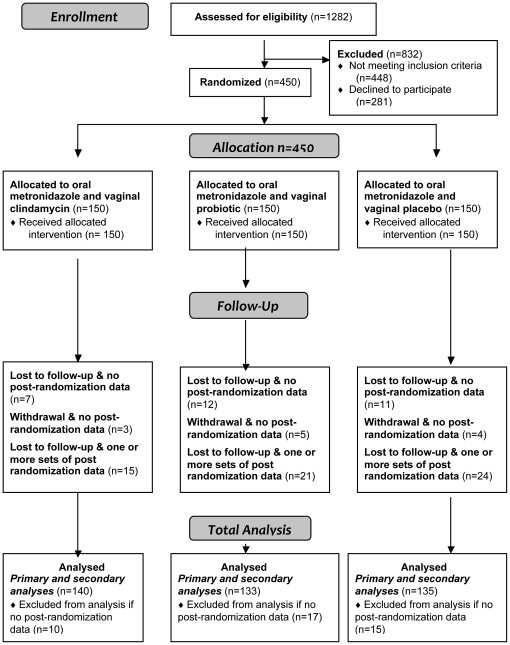
Participant Flow.

From November 2007 to May 2010, there were 1282 MSHC attendees with a diagnosis of BV, of which 448 (35%) were not eligible. Of the 834 remaining women with BV: 103 (12%) were not asked to participate, 281 (34%) declined and 450 (54%) agreed to participate, Figure 1. The demographic and behavioural characteristics of known eligible participants (n = 450) and non-participants (n = 384) were compared. Non-participants were more likely to be sex workers (32% versus 17%, p<0.01), to be Australian (66% versus 49%, p<0.01) and to report 100% condom use in the prior 3 months (15% versus 5%, p<0.01); indicating many non-participants relied on consistent condom use, and would be likely to have been deemed ineligible if referred to the research nurse. Of the 450 participants: 382 (85%) had a NS of 7–10 and 68 (15%) had 3–4 Amsel's criteria and a NS of 4–6.

All 450 participants were randomly assigned to and received their allocated treatment and no cross-over of treatments or imbalance in allocation occurred. There were no substantial differences between the three treatment groups in any demographic or behavioural characteristics, Table 1. Follow-up of participants occurred from November 2007 to December 2010, Figure 1.

**Table 1:** Basic demographic and behavioural characteristics of study population (n = 450:150 participants per arm).

	Treatment Arm of Clinical Trial		
Characteristic	Metro^a^/clinda^b^n (% or range)	Metro^a^/probiotic^c^n (% or range)	Metro^a^/placebo^c^n (% or range)
**Median Age (range) yrs**	27 (17–49)	27 (17–47)	27 (18–49)
**Ethnicity** d			
**Australian/British**	100 (67)	106 (71)	106 (72)
**Other**	50 (33)	44 (29)	42 (28)
**Educational level**			
**Primary &/or Secondary**	49 (34)	51 (34)	49 (35)
**Tertiary**	94 (66)	97 (66)	92 (65)
**Past history of BV**			
**No**	76 (52)	69 (46)	83 (58)
**Yes**	69 (48)	80 (54)	61 (42)
**Median Lifetime MSPs (range)**	11 (0–400)	12 (0–300)	10 (0–200)
**Median Lifetime FSPs (range)**	0 (0–50)	0 (0–40)	0 (0–66)
**Any new MSP last 12 months**			
**No**	39 (27)	47 (31)	40 (28)
**Yes**	104 (73)	101 (69)	102 (72)
**Any new FSP last 12 months**			
**No**	123 (85)	115 (79)	127 (86)
**Yes**	23 (15)	30 (21)	21 (14)
**Current RSP at time of randomization**			
**No**	38 (25)	55 (37)	53 (36)
**Yes**	112 (75)	94 (63)	96 (64)
**Current sex work**			
**No**	127 (85)	123 (82)	118 (89)
**Yes**	22 (15)	27 (18)	32 (21)
**Current smoker**			
**No**	83 (57)	74 (50)	83 (56)
**Yes**	62 (43)	75 (50)	65 (44)
**Current use of a hormonal method of contraception** e			
**No**	96 (64)	99 (66)	107 (72)
**Yes**	54 (36)	50 (34)	42 (28)

a Metro = oral metronidazole 400 mg twice daily for 7 days,

b clinda = one applicatorful of 2% vaginal clindamycin cream nightly for 7 nights,

c placebo and probiotic = vaginal pessary for 12 nights,

d Aus/UK ethnicity = identify as either Australian or British, other ethnicity comprised predominantly of individuals from South East Asia, China, Northern Europe and North America,

e hormonal contraception = oral contraceptive pill, implants such as implanon, Depo-Provera® and hormonal rings such as Nuvaring®, MSP = male sexual partner, FSP = female sexual partner, RSP = regular sexual partner. Missing data for specific variables for ≤5% of participants. Missing data excluded from the analysis and proportions are calculated from available data. Proportion with missing data did not differ between arms.

### Numbers analysed

Participants were analysed according to their randomized groups. Forty-two participants provided no post-randomization data were not included in analyses. The ***primary and secondary analyses*** therefore involved all participants providing post-randomization NS data [n = 408 (91%)], Figure 1. Of the 42 women who provided no post-randomization data, 30 were lost to follow-up and 12 withdrew; most gave no reason for withdrawal, although three stated they did not take medication. Those not included were equally distributed between groups (p = 0.35) and were more likely to be smokers (69% compared to 44%, p = 0.003), but there were no other significant demographic or behavioural differences (*data not shown* P>0.13). Fifty-seven participants (13%) were lost to follow-up before reaching 6-months or developing recurrence (NS = 7–10) and were equally distributed between groups; six month retention rates were therefore 78% (n = 351). These 57 participants all had a NS<7 at last observation and contributed person-time to the survival analyses as non-recurrent cases on the basis of their last Nugent score. The same 408 women were included in the s***econdary analysis*** where the endpoint was abnormal vaginal-flora (NS = 4–10).

### Concurrent medications and genital infections at baseline

Concurrent medications prescribed at baseline by referring clinicians occurred at similar rates between groups, Table 2. Fifty-two (13%) participants had *Candida* spp detected on culture and 23 (6%) on microscopy. STI screening of participants at baseline (94%) revealed 25 (7%) had chlamydia, and 2 (0.5%) had gonorrhoea, and none had trichomoniasis. There were no differences in STIs or candidiasis between the groups.

**Table 2:** Concurrent medications and self-reported adherence to trial medication (n = 408).

*Concurrent medications prescribed at baseline to participants*				
	Clindamycin Arm n = 140 (%)	Probiotic Arm n = 133 (%)	Placebo Arm n = 135 (%)	P value
**Oral fluconazole** *	23 (16.4)	22 (16.5)	21 (15.5)	0.97
**Doxycycline** *	4 (2.9)	2 (1.5)	4 (3.0)	0.69
**Azithromycin** *	18 (12.9)	10 (7.5)	18 (13.3)	0.25
**Ceftriaxone** *	2 (1.4)	1 (0.8)	3 (2.2)	0.70
**Antivirals**	2 (1.4)	4 (3.0)	2 (1.5)	0.61
**Other antibiotics**	3 (2.1)	7 (5.3)	5 (3.7)	0.36
**Vaginal antifungal**	2 (1.4)	1 (0.8)	0	0.66

*Self-reported adherence to oral and vaginal therapies by treatment group*				
	Clindamycin Arm n = 140 (%)	Probiotic Arm n = 133 (%)	Placebo Arm n = 135 (%)	P value
**Self reported adherence to metronidazole**				
**All/nearly all tablets taken**	127 (93)	120 (92)	124(92)	0.91
**≥50% tablets taken**	133 (97)	128 (99)	130 (96)	0.86
**No tablets taken**	1 (1)	1 (1)	1 (1)	1.0
**Self reported adherence to vaginal therapy**				
**All/nearly all product used**	121 (88)	100 (77)	104 (78)	0.04
**≥50% product used**	130 (95)	115 (89)	119 (89)	0.20
**No product used**	3 (2)	8 (6)	4 (3)	0.27

* Fluconazole prescribed as 150 mg stat dose, doxycycline as 100 mg bid for 7 days, azithromycin as 1 g stat dose and ceftriaxone as 500 mg IM.

### Self-reported adherence to trial medication and vaginal-product concealment

Self-reported adherence to oral metronidazole was high; ≥92% of participants took “all/nearly all" with no difference between study groups, Table 2. Self-reported adherence was higher to clindamycin (88%) compared to the other two groups (77–78%) when assessed as ‘all/nearly all’ (p = 0.04). Participants were asked to tick which vaginal product they thought they had received or “don't know" at completion of the trial; 87% of participants either did not know or incorrectly guessed the vaginal medication that they had received.

### Interim antibiotic use during follow-up in the community

Interim oral and vaginal therapies were commonly self-reported. Possible community-use of metronidazole (n = 10) was self-reported, none in the probiotic-group (0%) compared to 4.3% in the clindamycin and 2.9% in the placebo groups, p = 0.05, Table 3. Metronidazole was prescribed at MSHC for 17 participants with clinical features of BV but a NS of 4–6. Combined community and clinic metronidazole use (n = 27) did not differ between treatment groups, p = 0.49. There was no difference in reported use of vaginal treatments between treatment arms; the majority were antifungals, with clindamycin cream uncommonly reported.

**Table 3:** Interim antibiotic use and self-reported side effects to trial medications (n = 408).

*Self-reported antibiotic use in the community during follow-up*					
	Study Population n = 408 (%)	Clindamycin Arm n = 140(%)	Probiotic Arm n = 133 (%)	Placebo Arm n = 135 (%)	P value
**Oral metronidazole**	10 (2.5)	6 (4.3)	0	4 (2.9)	0.05
**Oral macrolide**	15 (3.7)	7 (5.0)	4 (3.0)	4 (2.9)	0.67
**Oral tetracycline**	5 (1.2)	3 (2.1)	1 (0.8)	1 (0.7)	0.63
**Oral penicillin or cephalosporin**	23 (5.6)	10 (7.1)	6 (4.5)	7 (5.2)	0.64
**Other unknown oral therapy** *	99 (24.3)	42 (30)	30 (22.6)	27 (20)	0.14
**Vaginal antifungal**	67 (17.0)	34 (24.3)	21 (15.8)	23 (17)	0.16
**Vaginal clindamycin**	2 (0.5)	1 (0.7)	0	1 (0.7)	0.77
**Other unknown vaginal therapy** †	19 (4.7)	10 (7.1)	5 (3.8)	4 (2.9)	0.27
*Interim metronidazole prescribed at Melbourne Sexual Health Centre during follow-up*					
**Oral metronidazole**	17 (4.7)	4 (2.9)	6 (4.5)	7 (5.2)	0.58

*Self-reported side effects to trial medication*					
	Study Population n = 408 (%)	Clindamycin Arm n = 140 (%)	Probiotic Arm n = 133 (%)	Placebo Arm n = 135 (%)	P value
**Vaginal itch/soreness**	123/378 (33)	41/130 (32)	43/121 (36)	39/127 (31)	0.82
**Nausea**	30/372 (8)	9/126 (7)	5/118 (4)	16/128 (13)	0.06
**Vomiting**	2 (0.5)	0	1 (0.8)	1 (0.8)	0.77
**Abdominal Pain**	4 (1.1)	1 (0.7)	1 (0.8)	2 (1.6)	1.0
**Diarrhoea**	1 (0.3)	1 (0.7)	0	0	1.0
**Headache**	7 (1.9)	5 (3.7)	1 (0.8)	1 (0.8)	0.21
**Metallic Taste**	9 (2.4)	5 (3.7)	3 (2.3)	1 (0.8)	0.23
**Body rash**	2 (0.5)	0	1 (0.8)	1	0.33
**Increased VD**	7 (1.9)	0	6 (4.7)	1 (0.8)	0.006
**VD containing medication**	4 (1.1)	1 (0.7)	2 (1.6)	1 (0.8)	0.70
**UTI**	3 (0.8)	0	2 (1.6)	1 (0.8)	0.32
**Possible PID**	1 (0.3)	0	0	1 (0.8)	1.0
**Genital HSV**	1 (0.3)	0	0	1 (0.8)	1.0

VD = vaginal discharge, UTI = urinary tract infection, PID = pelvic inflammatory disease, HSV = herpes simplex.

virus. Data available from the month one questionnaires for 367–395 (90–96%) participants for specific side effects. Proportion with missing data did not differ between arms. Denominators used to calculate proportions with side effects in each treatment group reflecting available and not missing data.

* Unknown oral therapies may have been antibiotics or oral antifungals and could not be verified,

† likely to have represented vaginal antifungal agents but could not be substantiated.

### Outcomes and estimation

#### Primary Outcomes

The 408 women included in the primary analysis contributed 112.5 person years (py) of follow-up. There was no difference in observed follow-up between the treatment groups (placebo 39.8 py, clindamycin 39.8 py and probiotic 43 py), p = 0.94. One month BV recurrence (NS 7–10) rates were 3.6%, 6.8% and 9.6% in the clindamycin, probiotic and placebo groups respectively, p = 0.13, table 4. Hazard ratios (HR) for BV recurrence at month were 0.40 (95% CI 0.14–1.10) for the clindamycin arm and 0.74 (95% CI 0.32–1.74) for the probiotic arm compared to placebo, p = 0.08. The clindamycin group was significantly more adherent to the vaginal product (when assessed as *taking all or most of the product*) compared to the probiotic and placebo groups, 88% compared to 77–78% (p = 0.04). Neither treatment group was significantly more effective at reducing BV recurrence at one month than the placebo group, after adjustment for adherence to the vaginal product, [clindamycin HR = 0.43 (95% CI 0.15–1.22) and probiotic HR = 0.75 (95% CI 0.32–1.76)].

**Table 4:** Recurrence Rates and Relative Risk of Recurrence of BV and of Abnormal flora at Month one and Month six by Treatment Group (n = 408)†.

*Primary Study Outcomes by Treatment Arm*				
	Treatment Arms			
	Clindamycin n = 140(%, 95%CIs)	Probiotic n = 133(%, 95% CIs)	Placebo n = 135(%, 95% CI)	Study Population n = 408(%, 95% CI)
**M1 BV recurrence rate** *	5/140 (3.6, 1.3–7.7)	9/133 (6.8, 3.4–12.1)	13/135 (9.6, 5.4–15.5)	27/408 (6.6, 4.5–9.4)
**Hazard ratio for M1 BV recurrence compared to placebo** ‡	0.43 (0.15–1.22)	0.75 (0.32–1.76)	-	**-**
**Cumulative M6 BV recurrence rate** *	42/140 (30.0, 22.8–38.0–38)	37/133 (27.8, 20.7–35.9)	36/135 (26.7, 19.7–34.6)	115/408 (28.2, 24.0–32.7)
**Hazard ratio for cumulative M6 BV recurrence compared to placebo** ‡	1.09 (0.69–1.70)	1.03 (0.65–1.62)		

*Secondary Study Outcomes by Treatment Arm (n = 408)* *				
	Treatment Arms			
	Clindamycin n = 140(%, 95%CIs)	Probiotic n = 133(%, 95%CIs)	Placebo n = 135(%, 95%CIs)	Study Population n = 408(%, 95%CIs)
**M1 abnormal flora recurrence rate** *	32/140 (22.9, 17.4–29.1)	29/133 (21.8, 15.4–29.4)	33/135 (24.4, 17.8–32.2)	94/408 (23.0, 19.1–27.3)
**Hazard ratio for M1 abnormal flora recurrence compared to placebo** ‡	0.98 (0.60–1.62)	0.97 (0.59–1.60)		
**Cumulative 6-month abnormal flora recurrence** *	78/140 (55.7, 47.4–63.8)	70/133 (52.6, 44.1–61.0)	72/135 (53.3, 44.9–61.6)	220/408 (53.9, 49.1–58.7)
**Hazard ratio for M6 abnormal flora recurrence compared to placebo** ‡	1.00 (0.72–1.38)	0.96 (0.69–1.33)		

M1 = month one, M6 = month 6,

† missing data included in the denominator in calculation of recurrence rates- month one data missing on 44 women equally distributed between treatment arms (13,18,13) and month six data missing on 57 women equally distributed between treatment groups (14,22,21),

* chi square test for comparison of BV recurrence rates between the three treatment groups at month one (p = 0.13) and month six (p = 0.82) and for comparison of abnormal flora recurrence rates between the three treatment groups at month one (p = 0.87) and month six (p = 0.87).

‡ All Hazard ratios at month one and six for BV and abnormal flora recurrence were adjusted for adherence to the vaginal product as indicated in the results.

**Figure 2:**
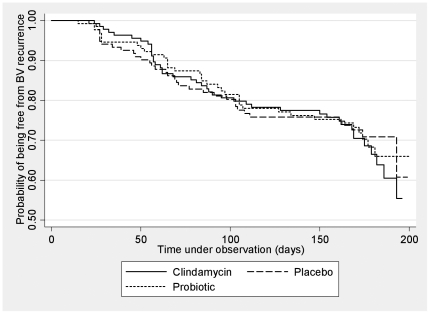
Probability of being free from BV recurrence over 6 months.

At six months, there was an overall cumulative BV recurrence rate of 28.2% (24.0–32.7%) and no difference between the treatment arms (p = 0.82; Table 4). Figure 2 depicts the Kaplan-Meier survival analysis with no difference between treatment groups (log rank test, p = 0.93). The unadjusted HR for BV-recurrence for vaginal clindamycin and the vaginal probiotic compared to placebo were 1.09 (95% CI 0.70–1.70) and 1.02 (95% CI 0.64–1.61), respectively. When adjusted for adherence to vaginal therapy as previously defined, the HRs for BV recurrence at six months compared to placebo were unchanged at 1.09 (95% CI 0.70–1.70) for vaginal clindamycin and 1.03 (95% CI 0.65–1.63) for the vaginal-probiotic.

#### Secondary Outcomes

**Figure 3:**
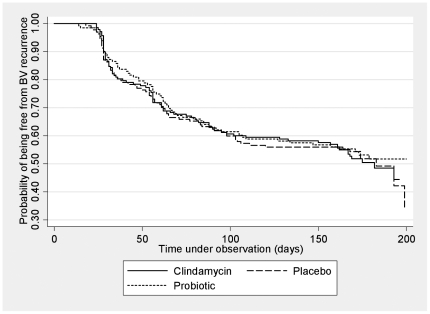
Probability of being free from abnormal flora recurrence over 6 months.

Four hundred and eight women were included in the secondary analysis. One month and cumulative 6-month recurrence rates for abnormal vaginal-flora (NS 4–10) were 23% (95%CIs 19–27%) and 54% (95% CIs 49–59%), respectively, and did not differ between treatment groups (p = 0.87; Table 4). The HRs for one and six month BV recurrence, adjusted for adherence to vaginal therapy, are shown in table 4, and at each time point neither treatment group was superior to placebo. Figure 3 depicts the Kaplan-Meier survival analysis, again with no difference between treatment groups (log rank p = 0.96).

### Adverse Events

Self-reported adverse effects are shown in Table 3. One third of participants reported vaginal itching/soreness and 8% reported nausea; all other side effects were uncommon or rare and the only adverse symptom that significantly differed between treatment groups was increased vaginal discharge, which was reported by more participants in the probiotic group (p = 0.006).

## Discussion

This RCT shows that 7 days of oral metronidazole combined with 2% vaginal clindamycin cream or a 12 day course of a commercially- available vaginal *L. acidophilus* probiotic does not achieve higher cure rates for BV compared with oral metronidazole monotherapy over six months of follow-up. These findings suggest that combining first line regimens and achieving broader spectrum antibiotic coverage does not improve the clinical outcome above a standard course of oral metronidazole.

This is the first placebo-controlled RCT to evaluate the effect of combining oral metronidazole with vaginal clindamycin. These regimens have been shown to have equivalent clinical efficacy as monotherapy with one month cure rates of 80% [3], despite differing spectrums of antimicrobial activity. Metronidazole is active against anaerobes, whereas clindamycin has broader spectrum activity against Gram-positive aerobes and anaerobes. The microbial flora pattern following treatment with clindamycin differs to metronidazole, with greater reduction in *Mobiluncus* spp and higher frequency of clindamycin-resistant anaerobes [14], [15], [16]. Meltzer also confirmed the relative resistance of *Mobiluncus* spp to metronidazole therapy, and showed their persistence was associated with increased risk of BV recurrence [17]. Based on these data and the polymicrobial nature of BV, one might postulate that higher cure rates could be reached by combining metronidazole and clindamycin. The combination has the potential to achieve i) broader spectrum activity against possible aetiological agents and other BV-associated bacteria that may contribute to symptomatology and sequelae, ii) reduction in the selection of macrolide-resistant species, and iii) high concurrent vaginal and systemic levels of antibiotic to promote more effective clearance of BV-associated bacteria. Conversely, combination therapy could have resulted in higher rates of adverse effects. We found no therapeutic benefit from combining first line therapies, but also no increase in adverse symptoms, particularly symptomatic candidiasis, in the combination antibiotic arm. Importantly, ongoing analysis will assist in determining whether any behavioural factors play a significant role in recurrence following treatment.

This trial also tested the efficacy of combining a 12-day course of a commercially available vaginal-probiotic containing ≥10^7^ CFU of live *L. acidophilus* and 0.03 mg oestriol with oral metronidazole, and found no benefit over metronidazole monotherapy. This product demonstrated improved restoration of normal vaginal-flora in two studies in BV-positive women [6], [7]. In a placebo-controlled RCT of 32 women diagnosed with BV by the Amsel method, Parent found 6 nights of the probiotic achieved two and four week cure rates of 77% and 88% compared to 25% and 22% in the placebo group, respectively, p<0.05 [7]. A statistically significant increase in *Lactobacillus* spp on Gram-stain was recorded in the treatment compared to the placebo group at two and four weeks, p<0.05. In a randomized-blinded placebo-controlled trial of 366 women, Ozkinay reported a statistically significant improvement in the normal-flora-index (a composite of lactobacillus count, vaginal pH, leucocytes and pathogenic micro-organisms) of women with BV, candidiasis or trichomoniasis following anti-infective therapy and 12 nights of vaginal-probiotic compared to placebo, p<0.002 [6].

Probiotic therapies are emerging as popular over-the-counter treatments for vaginal candidiasis and BV. As loss of *Lactobacillus* spp in the vagina is a characteristic of BV, it is thought that lactobacillus-probiotics may support restoration of the normal lactobacillus-dominant state. Several vaginal *Lactobacillus* spp have host protective characteristics, including hydrogen peroxide, lactic acid and biosurfactant production, antimicrobial activity, and coaggregation with pathogens [18], [19]. While a considerable number of probiotic studies have been conducted, the lactobacillus species in the probiotics have varied substantially in their characteristics, their ability to colonise the vagina [20], and adherence to vaginal epithelial cells [21]. A 2009 Cochrane review found that only 4 RCTs, including the trial by Parent [7], met the inclusion criteria for a systematic review [5], and substantial heterogeneity in products, trial methodologies and outcome measures meant that there was insufficient evidence to establish the role of probiotics in BV. More recent observational data using prolonged repetitive courses of lactobacillus-probiotics appear to show more promise than short courses [22], [23], [24]. Another important advance in the field has been the development of a probiotic containing a human hydrogen-peroxide producing *L. crispatus*, which appears acceptable and safe in early studies [25], [26]. However, large-scale well-designed clinical trials with standardized outcomes to enable direct comparison between probiotics is a priority to advance the field [5].

An important limitation of this trial was that the products and formulations could not be identical in duration and appearance. Clindamycin ovules are not available in Australia so investigators used the cream. The probiotic could only be obtained as a pessary and the decision was made to match the placebo to this pessary. Considerable care was therefore taken with concealment of the nature of the vaginal therapy. Participants were not given any trade-names or information to enable them to link a product with its appearance or duration of therapy, and the placebo could not be distinguished from the probiotic. Trial medications were in plain packaging, sealed in opaque boxes and participants were instructed to open their kits at home. No study staff had access to the randomization schedule and the primary and secondary outcome of the trial was the Nugent score, determined by blinded microscopists with no access to any other trial data. We considered 87% of participants not knowing or incorrectly guessing which vaginal medication they had received as evidence of successful concealment.

Another important aspect of the trial was that the Nugent method was used to assess the endpoint of BV as it is widely considered to be the most objective diagnostic measure, is relevant and generalizable to clinical trials, and is suited to extended follow-up in the community. The limitation of this approach is that some participants with symptoms of BV, but intermediate flora, were considered as non-recurrent on the basis of the strict Nugent criteria defined primary outcome (NS 7–10); these cases were counted as recurrent cases in the secondary analysis of abnormal flora. The use of the Nugent method is therefore likely to result in an under-estimation of the recurrence rate of clinically-evident BV, as a proportion of women with intermediate flora will have BV on the Amsel criteria. In this trial, interim metronidazole use for BV symptoms accompanied by intermediate vaginal flora (n = 17), or probable metronidazole use in the community without Nugent score data (n = 10), occurred in 27 participants over 6 months of follow-up, and did not differ by treatment group (p = 0.49). Participants were encouraged to contact us with interval symptoms, to facilitate interim access and additional sample collection; however, the inability to capture all clinically relevant episodes of BV using the Nugent method may have under-estimated the clinical recurrence rate in each arm by up to 4–8%. Importantly, the aim of this study was not to determine the clinical recurrence rate of BV, but to determine if the recurrence rates differed between treatment groups, and participants with clinically relevant symptoms not captured by the primary outcome measure did not differ between treatment groups. Lastly, 408 (91%) participants provided one or more Nugent scores following treatment and contributed person-time to survival analyses. However, the six month retention rate for the trial was 78%. This 22% loss to follow-up over six months may have potentially reduced our statistical power to detect a small treatment effect but this loss to follow up was evenly distributed between the study groups.

The strengths of our trial include that it was a large RCT with sufficient power to detect a clinically meaningful difference in efficacy between the treatment groups. The groups were well-balanced for demographic and behavioural characteristics at baseline, and we collected detailed epidemiological data to determine if behavioural characteristics are associated with recurrence, which is the subject of future planned analyses. We applied the gold standard Nugent method to all slides to ensure generalizability of our findings, and have an ongoing quality assurance programme to ensure consistency between our experienced microscopists. We performed extended follow-up for six months with frequent evaluations which would have detected early non-sustained differences between the arms if they had occurred. High levels of adherence were reported to oral and trial medication.

This trial shows no additional benefit from combining oral metronidazole with vaginal clindamycin cream or a commercially available vaginal-*L.acidophilus* probiotic. Current internationally recommended treatments are delivered as monotherapies, but have not achieved high sustained cure rates whether used for a week or in suppressive or periodic presumptive regimens. There is a well-recognized need for more effective management of both initial and recurrent BV, both to alleviate symptoms and to reduce its adverse the sequelae. How integral behavioural factors are in the development and recurrence of BV is still not completely understood, and remains an important area of research to advance our management of this common condition.

## Supporting Information

Checklist S1 CONSORT Checklist.
(DOC)

Protocol S1 Trial Protocol.
(RTF)
