# Supplementary material for: Efficacy of Oral Metronidazole with Vaginal Clindamycin or Vaginal Probiotic for Bacterial Vaginosis: Randomised Placebo-Controlled Double-Blind Trial
Source: PLoS One. 2012 Apr 3;7(4):e34540. doi: 10.1371/journal.pone.0034540 (PMC3317998; doi:10.1371/journal.pone.0034540)
Supplement: Protocol S1 — Trial Protocol. (RTF) [file pone.0034540.s002.rtf]

AIM
To determine if the addition of either topical clindamycin or a topical probiotic/oestrogen combination, reduces recurrent rates of bacterial vaginosis (BV), when added to the current recommended therapy of seven days of oral metronidazole.

BACKGROUND
Summary Points
·	Bacterial vaginosis (BV) is one of the commonest genital infections (point prevalence of 10-30%) in women of reproductive age.
·	BV is associated with serious sequelae, including the facilitation of transmission of HIV and sexually transmissible infections (STIs) and adverse pregnancy outcomes. 
·	The population attributable risk of BV for preterm delivery in the USA is estimated to be 30%, at a cost of USD 1 billion per annum [1]. 
·	The population attributable risk of BV for antenatal HIV transmission in a high prevalence population in Africa is 23% [2].
·	Current recommended treatment for BV is mono-therapy with seven days of oral metronidazole or vaginal clindamycin. 
·	We recently conducted the first 12 month follow up study after seven days of oral metronidazole and showed that 58% of women experienced recurrence of BV and 69% had recurrence of abnormal flora 
·	Improved regimens for the treatment of BV are urgently needed and may result in significant public health benefits, such as a reduction in pre-term delivery and HIV transmission.
·	Combination antibiotic therapy may be more effective than mono-therapy in the treatment of this polymicrobial syndrome.
·	Replacement of Lactobacillus species (spp) to restore vaginal colonisation and an acidic vaginal pH has also been identified as a promising therapeutic approach for BV. 
·	We are conducting the first randomised trial to evaluate dual antibiotic therapy (oral metronidazole and vaginal clindamycin) and oral metronidazole and a vaginal probiotic with oestrogen (Lactobacilli spp and oestriol) against recommended mono-therapy (7 days of oral metronidazole) for the treatment of BV.

Bacterial vaginosis, the condition
Bacterial vaginosis (BV) is the commonest cause of abnormal vaginal discharge in women of reproductive age, but despite an increasing understanding of the pathogenesis and sequelae, the precise aetiology remains unknown. BV is a clinical syndrome characterised by a disturbance of normal vaginal flora [3], with a loss of H202 producing Lactobacillus species (spp.), and an increase in Gram variable coccobacilli (Gardnerella vaginalis and Bacteroides spp), anaerobic organisms (Mobiluncus spp, Fusobacterium spp, Prevotella spp and Peptostreptococcus spp) and genital mycoplasmas (Mycoplasma hominis & Ureaplasma urealyticum). Associated with these changes in flora, there is a rise in vaginal pH, and increased production of proteolytic enzymes, organic acids and volatile amines [4]. This condition occurs in sexually active pre-menopausal women and results in an abnormal vaginal discharge and an unpleasant odour. Recurrence following recommended therapy is common.  

BV is one of the commonest genital conditions in women of reproductive age
The point prevalence of BV varies according to the population studied, but ranges from 9-30% in pregnant women, to 20-40% in females attending sexual health services, to in excess of 50% in women in sub-Saharan Africa [5]
Although there is little community-based data, in a UK study of 287 women presenting for Pap smears, 9% had BV and 14% abnormal flora [6]. A US study of 13,747 women attending medical services between 23-26 weeks gestation found an overall prevalence of 16%; 23% of African American women had BV compared to 9% of white Americans [7]. 

BV is associated with significant symptoms and serious sequelae
BV is symptomatic in up to 50% of laboratory-diagnosed cases. Symptoms are characterised by a malodorous and often profuse vaginal discharge, which causes considerable distress and discomfort to affected women. It is associated with serious sequelae, such as chorioamnionitis, spontaneous abortion [8-10], preterm delivery and low birth weight [8, 11, 12], post-partum and post-abortion endometritis, post hysterectomy vaginal-cuff infection and increased susceptibility to HIV and sexually transmitted infections (STIs) [2, 13-15]. It is estimated that the population attributable risk of BV for preterm delivery in the US is 30%, at a cost of USD 1 billion per annum [1]. BV increases HIV transmission in both case-control and cohort studies (relative risk ranging from 2-4 fold) [2, 8, 10, 11, 13], with an attributable risk for antenatal HIV seroconversion of 23% in a high prevalence population of pregnant women in Malawi [2]. As BV is most prevalent in populations at risk of HIV, identifying more effective therapies for BV is integral to effective HIV control. 
Current recommended mono-therapies for BV have poor efficacy
Current treatment for BV is directed towards restoring the normal vaginal ecology as the cause of the syndrome is unclear.  Current internationally recommended therapies are therefore primarily aimed at eradicating anaerobic flora in BV and include oral metronidazole or vaginal clindamycin [16]. These agents have equivalent one month efficacy in seven day regimens with cure rates of 70-90% [17], but comparative studies with follow-up beyond 3 months have not been undertaken. We recently conducted the first large prospective study of recurrence of BV over 12 months after oral metronidazole, and found recurrence rates that were unacceptably high: abnormal flora (69%) and BV (58%) (Figure 1) [18].  Our findings indicate that current recommended mono-therapy with metronidazole provides only short- term relief and is associated with high rates of long-term recurrence.  

Figure 1: Kaplan Meier survival curve demonstrating recurrence of bacterial vaginosis over 12 months following treatment with 7 days of oral metronidazole (n=130). Dashed lines indicate 95% confidence intervals.


Rationale for combination therapy 
Although BV recurrence rates are high, a proportion of women (approximately 30%) do appear to respond to current mono-therapies at 12 months [18], suggesting at least partial efficacy of these agents. Recent studies using culture-independent molecular methods, such as 16S rDNA PCR, have confirmed that women without BV have a homogenous lactobacillus-dominant vaginal flora, whereas the composition of vaginal flora in BV is heterogeneous with high species diversity and high bacterial loads [19, 20]. It is possible that current therapies are providing insufficient coverage against the broad range and high load of organisms present in this polymicrobial syndrome. Therefore, we are conducting a trial of dual therapy for BV using a sequential combination of currently recommended first-line therapies. Clindamycin and metronidazole differ in their spectra of activity: the former is active against anaerobic, aerobic and facultative bacteria, and the latter against a broad-range of anaerobes. Combination of these two agents will be more likely to cover the diverse range of organisms present in BV, and delivery by both oral and vaginal routes will achieve high vaginal concentrations of both agents but minimize systemic effects.

Rationale for the use of probiotics and evidence of their effectiveness.  
While this trial will be comparing recommended monotherapy (metronidazole) to dual therapy with metronidazole and clindamycin, we also plan to include a third arm in this study to determine if replacement of Lactobacillus spp concurrently with metronidazole will improve recurrence rates compared to standard therapy. The probiotic being used, Gynoflor®, also contains oestriol. The rationale for the use of this particular combination is discussed below. 

As the aetiology of BV remains unclear, an alternative or additional approach to antibiotic therapy has been the use of Lactobacillus spp. The reasoning behind this approach is that Lactobacillus spp are the dominant micro-organism in the healthy human vagina [21] and depletion or loss of this species is consistently seen as part of the syndrome of BV [4, 22]. Lactobacillus species perform several roles which are integral to vaginal defence [23]: they maintain an acidic vaginal pH by metabolising glucose to lactic acid, many species manufacture hydrogen peroxide, which has antimicrobial activity [24], and lactobacilli produce bacteriocins and bacteriocin-like substances which inhibit other microbes [23]. Whether the presence of high loads of anaerobic and mixed flora directly results in the loss of lactobacilli or another agent is responsible is unknown [25]. 

Small studies using non-vaginal Lactobacillus species and even vaginal acidifying agents, indicate that restoration of a normal vaginal pH may be sufficient to enable recovery of normal vaginal flora [26-28].

Gynoflor and the evidence it may be beneficial in the treatment of BV
Gynoflor is a preparation containing Lactobacillus acidophilus and 0.03mg oestriol, which has been approved for use for the treatment of BV in many European countries and is manufactured in Switzerland (Medinova, Switzerland).  Two randomised trials (RCTs) have evaluated Gynoflor in the treatment of BV.  The first RCT by Parent et al evaluated Gynoflor treatment for six days without antibiotics in 32 pre-menopausal women with BV, and assessed BV recurrence using Amsel's criteria.  Ten of the Gynoflor group (77%) achieved BV cure at 14 days by Amsel criteria compared to only 3 (25%) in the placebo group (p<0.01) [29].  The intervention group also had significantly higher rates of vaginal colonisation with lactobacilli than the placebo group (88% versus 14%).  This study was promising but had a number of important limitations: follow up was limited to only 28 days, Gynoflor was not used with a recommended antibiotic treatment, the Amsel method which is a less objective method than the Nugent method was used to evaluate the women, and there was a 47% loss to follow-up (15/32). 

In the second RCT by Ozkinay et al, 360 women with a diverse group of vaginal infections were randomised to receive either Gynoflor for six days or placebo, two to seven days after the appropriate antibiotic therapy was completed [30]. The Normal Flora Index (NFI) was significantly better in the study group: p=0.002 at 4-7 days and 0.006 at 4-6 weeks. Unfortunately, BV was diagnosed at enrolment in only 19 participants, results were not reported by initial diagnosis and a non-standard diagnostic method for BV was used. Importantly, Gynoflor was well tolerated with only one woman in the intervention group reporting diarrhoea. 

There are four other published trials that evaluate the use of probiotics for treatment of BV. One trial used the “Ellen tampon”, a tampon impregnated with several species of Lactobacillus, following vaginal clindamycin therapy [31]. There was no improvement in the BV cure rate after treatment with lactobacilli-containing tampons compared to placebo tampons. Another double blind, placebo-controlled treatment trial with lyophilized Lactobacillus acidophilus, but no antibiotic therapy, also demonstrated no benefit. [32]. Two early trials used yoghurt with Lactobacillus acidophilus [33, 34], but had many methodological flaws in design and therefore results are of uncertain significance. In Shalev's study which used a cross-over design, only 7 women completed the protocol and were included in the analysis [33]. The other trial was open label, with patients refusing treatment acting as controls [34]. 

In summary, the two Gynoflor studies provide some evidence to indicate that Gynoflor may improve the cure rate in BV and importantly they demonstrate that it is safe and well tolerated.  We plan to use recommended therapy (seven days of oral metronidazole) to achieve a rapid reduction in the high concentrations of anaerobic flora present in BV and to concurrently us a Lactobacillus-containing probiotic with the intention of restoring local colonisation and an acidic vaginal pH after eradication of BV-associated organisms. We anticipate this approach is likely to be more successful in restoring normal vaginal flora than the use of probiotics alone. Gynoflor also contains oestriol, the rationale behind inclusion of oestrogen is discussed below. 

Rationale for the inclusion of oestrogen and evidence for hormonal modulation of BV
Women of reproductive age have a lactobacillus-dominant flora and an acidic vaginal pH (<4.5), in contrast to pre-menarchal and post-menopausal females where the vaginal flora predominantly consists of anaerobic rods and cocci and vaginal pH is more alkaline (>4.5). The vaginal microflora of women is directly related to oestrogen levels [4]. Oestrogen increases deposition of glycogen in vaginal epithelial cells and glycogen is a substrate for Lactobacillus spp, which generate lactic acid from glucose [4]. Lactic acid has been shown to be a potent inhibitor of BV-associated organisms [35]. Cross-sectional studies, including our own, have also shown that the use of hormonal contraception is associated with a lower risk of BV [35-37]. Our recent cohort study provided the only published prospective data indicating that hormonal contraception may be associated with a lower risk of recurrent BV [18].  Topical vaginal oestrogen preparations are commonly used in post-menopausal women and oestrogen-deficient states to improve vaginal dryness, they are well tolerated and minimal systemic absorption occurs so that they are safe in conditions where oral oestrogen preparations are contraindicated. We anticipate an added benefit from the inclusion of oestriol in Gynoflor on the vaginal pH and flora.
Public health implications of BV
BV is common and is associated with significant sequelae. Perhaps the two greatest areas of public health significance are its association with adverse pregnancy outcomes, specifically preterm delivery, and its association with increased transmission of HIV. In addition, as the most common cause of abnormal vaginal discharge, significant time and health resources are spent in diagnosis and treatment of this condition. While current treatment regimens have poor efficacy, it will be difficult to achieve significant reductions in BV-associated sequelae, and considerable clinical resources are consumed by repeated presentations of women with BV recurrence to medical services. If more than half of treated women have experienced BV recurrence within a year of recommended therapy, clearly there is a need to investigate alternative more effective approaches to the management of this common condition.
RESEARCH PLAN
Aims 
The primary aim of this study is to determine if the use of combination oral metronidazole and vaginal clindamcyin therapy and/or the combination of oral metronidazole with vaginal probiotic/oestrogen therapy is associated with lower recurrence rates of BV at six months than the current recommended therapy for BV oral metronidazole alone.
Secondary aims are to investigate behavioural practices and other factors associated with BV recurrence following therapy.

Hypotheses
1.	Combination antibiotic therapy of oral metronidazole with vaginal clindamycin will be more effective than mono-therapy in the treatment of bacterial vaginosis.
2.	Restoring local vaginal flora and an acidic vaginal pH through replacement of Lactobacillus spp and topical oestrogen (in addition to oral metronidazole), will achieve higher rates of cure than current recommended therapy oral metronidazole alone.

Study design
Randomised double-blind placebo controlled trial for treatment of BV with 3 arms: oral metronidazole/vaginal placebo; oral metronidazole/vaginal clindamycin; and oral metronidazole/vaginal probiotic Gynoflor (Lactobacillus species and oestriol). 

Intervention
This trial has been designed so that all participants receive standard first-line therapy for BV in Phase 1 – i.e. 7 days of oral metronidazole (see table below). 

Intervention	Phase 1: Oral treatment 	Phase 2: Vaginal treatment 	Phase 3: follow up	
Arm one	Metronidazole 	Clindamcyin 	Follow up at 1, 2, 3, and 6 months	
Arm two 	Metronidazole 	Gynoflor 		
Arm three	Metronidazole 	Placebo 		

In Phase 2, the vaginal phase, to be undertaken concurrently with Phase 1, one third of participants will be randomised to receive 7 days of vaginal clindamycin cream (2%), one third will receive 12 days of a vaginal probiotic ovule Gynoflor, and one third will receive a vaginal placebo ovule (12 days of the placebo ovule). This is a double-blinded trial and neither interventions nor placebo will be identifiable by their packaging. 

Gynoflor  is a vaginal ovule containing at least 107 colony forming units of live Lactobacillus acidophilus, 0.03mg oestriol and 600mg lactose, manufactured and distributed in Europe by Medinova of Switzerland. The company-manufactured placebo preparation is identical to the study intervention, apart from containing no lactobacillus or oestriol.  Gynoflor and placebo will be provided free of charge. The Australian Therapeutic Goods Administration (TGA) uses the Clinical Trial Notification (CTN) Scheme to administer the use in clinical trials of products not currently entered on the Australian Register of Therapeutic Goods. 2% clindamycin cream is available in Australia and registered with the TGA for use in BV. 

The specific trial design has been developed to accommodate the different formulations and the differing durations of therapy of the two interventions being tested, while maintaining blinding of participants, researchers and clinicians. Importantly, it will be explained to all participants that they will receive either a vaginal cream or ovule for 7 or 12 days, but participants will not be informed whether clindamycin, Gynoflor or the placebo is in ovule or cream form to maintain blinding. 

Women will be instructed to use all vaginal treatments during menstrual periods.  When menses are particularly heavy, they may choose to omit vaginal therapy those days, but to ultimately take the full treatment regimen.  This information will be carefully recorded in addition to self-reported adherence. 

Participants
Women will be recruited for this study from the Melbourne Sexual Health Clinic (MSHC). The MSHC is the largest sexual health service in Victoria, and diagnoses 450 cases of BV annually. We have recently conducted successfully a 2 year longitudinal study of BV at MSHC [18].
  
Women will be eligible women if they are
·	pre-menopausal (18-45 years of age)
·	able to provide informed consent in English. 
·	have symptomatic BV defined by a Nugent score of 7-10 or 3-4 Amsel criteria with a Nugent score of 4-6. Nugent method is the standard diagnostic method for the diagnosis of BV. A Nugent score (NS) of 0-3 is classed as normal flora, 4-6 as intermediate flora and 7-10 as BV.
Exclusion criteria:
·	pregnancy
·	known HIV positive status
·	oestrogen-dependent tumors (breast, uterus, vagina)
·	abnormal vaginal bleeding of unknown aetiology
·	hypersensitivity or allergy to metronidazole, clindamycin or any of the ingredients of Gynoflor

Procedures
All women attending MSHC with vaginal symptoms are assessed for BV using the Nugent method by clinicians as part of their standard care. MSHC has an electronic alert that will trigger whenever clinicians enter the diagnosis of BV into the computerised record, to remind them to offer enrolment in the study to eligible women.  If a woman is interested in participating in the trial, informed consent will be sought by the designated research nurse, who will have been trained in this procedure.

Enrolled women will be randomised to one of the three arms of the study in equal proportions (one third in each arm) using a computer-generated randomisation sequence (see below). A computerised script will be sent to the on-site pharmacy where the treatment kit will be collected by the participant. The contents of the kit will be packaged so as to preserve blinding of clinic staff. Clear instructions on the administration of the oral and vaginal preparations and durations of therapy for each individual will be inside each kit. The participant will collect this kit from pharmacy staff at the end of the visit just prior to departure. Importantly, participants, clinicians and pharmacy staff will not be aware to which arm the participant has been allocated at enrolment. Participants will be reviewed and examined 7-14 days following completion of therapy. It is possible that women may inform a practitioner that they had used a vaginal ovule or cream, potentially identifying the clindamycin arm only but not differentiating between the probiotic and placebo. Due to this small risk of unblinding, assessment of the major trial outcome of BV will only be made using a laboratory diagnosis, as outlined below, not a clinical diagnosis. Laboratory staff, who do not have access to the clinical history, will score vaginal flora (the outcome measure for the study), therefore maintaining blinding. 

Visit procedures
Visit one (at enrolment);
All participants will have a clinical history taken and undergo speculum vaginal examination, including pH estimation (Spezialindikator strips pH 2-9, Merck & Co., NJ, USA).  After explanation of the trial, women will be invited to participate and to provide written informed consent. Participants will then be asked to complete a questionnaire (regarding behavioural practices, risk factors for STIs, symptomatology {rating scale 1-5}, previous BV episodes and prior treatments). The clinicians will complete a pre-formatted data collection sheet on examination findings (e.g. presence of discharge, nature of discharge), genital specimens will be collected for vaginal microscopy, Chlamydia trachomatis PCR, Neisseria gonorrhoeae culture, and Trichomonas vaginalis culture, and a separate vaginal swab will be stored at -70oC for later analysis.  Following randomisation, study medication will be dispensed as outlined.  

Follow up at 4, 8 weeks, 12 weeks and 6 months
Kits containing a questionnaire, vaginal swab, glass slide, instructions for self-collection, and a prepaid envelope, will be used to examine BV recurrence and factors associated with recurrence at 4, 8 weeks, 12 weeks and 6 months following treatment. The first kit for the 4 week follow-up will be given to participants at the enrolment visit, at which point clear verbal and written instructions will be also be provided and participants given the opportunity to clarify any concerns. Kits for follow-up at 8, 12 weeks and 6 months will be posted to participants. At each follow-up point, participants will be asked about sexual and contraceptive practices, genital symptoms, oral and vaginal self-treatments, and clinician-prescribed therapies. They will self-collect a vaginal swab and make a smear on a glass slide, and return the swab, slide and questionnaire in the prepaid envelope (18). Reminders in the form of telephone calls, letters or SMS text-messages will be sent once she has received the kit by post and weekly after the due date for four weeks. If no specimens are received by this time, the woman will be classified as a non-responder for that interval. Non-responders will still be contacted at the subsequent follow-up point(s). 

Interval BV symptoms and unscheduled visits
Women will receive a card and refrigerator magnet with the free call study telephone number, which will be staffed by the study nurse and research investigators. Participants will be encouraged to call the number if they have symptoms of BV during the study or concerns regarding treatment or possible side effects. A sample kit will be supplied to women so that if they are unable to attend MSHC they will be able to self-collect samples for interval symptoms after discussion with the research nurse. Women will be asked in the initial consenting process to allow any results from outside clinics for investigation of BV symptoms to be made available for the study. 

These procedures are similar to those we have previously used in the study of BV recurrence (18,36) and the trial of probiotics for vaginal candidiasis (38).  Only 3/130 and 3/287 women attended outside clinics in these studies as a result of providing easy and rapid review if a woman developed interval symptoms.

Stored samples
With participant consent, vaginal swabs will be stored at -80oC for future examination for potential pathogens. DNA will be extracted using the Magnapure system: MagnaPure LC (Roche Molecular System, Alameda, CA, USA) in accordance with manufacturer's protocol, followed by detection of potential pathogens using molecular amplification technology. 

Study Outcome Measures
Primary outcome measure
Recurrence of BV within 6 months will be defined as a Nugent score of 7-10. We have chosen 6 months rather than 12 months, based on our experience of relapse rates in our previous studies of BV (Figure1). Relapse of BV plateaus by 6 months. Additionally, compliance with follow up will be higher with a 6 month compared to 12 month follow up. 

Secondary outcome measures
1.	Recurrence of abnormal flora within 6 months will be defined by a Nugent score 4-10. 
2.	Behavioural practices and other factors (eg contraceptive use, past history of BV, smoking)  associated with BV recurrence.

Validation of primary outcome
All slides will be stored and re-scored by two independent experienced microbiologists blinded to the intervention group of the participant.  If slides vary in their Nugent score groupings (0-3, 4-6 and 7-10), our experienced laboratory staff will reach consensus using methods from our previous studies of BV recurrence (18, 36). 

Sample size calculation
The Melbourne Sexual Health Centre (MSHC) is the largest sexual health service in Victoria, and diagnoses 450 cases of symptomatic BV annually.  We estimate a BV recurrence of 50% in study arm 3 (placebo).  A sample size of 123 will be required in each group to detect a 20% difference in recurrence rates (recurrence rate of 30%) between study arm 1 or study arm 2 and study arm 3 with a power of 90% and an alpha of 0.05. Assuming a loss to follow up rate of 20%, 150 women will be recruited in each group. This would involve recruiting 50% of eligible women at MSHC which is consistent with previous studies given a research nurse will be on site to assist.

Evidence of past successful recruitment
Our team has previously successfully recruited women into similar trials.  In a cohort study of BV, we recruited 157 women from MSHC with BV in 12 months despite more restrictive inclusion criteria and no research nurse available to assist with recruitment (18, 36).  Investigators have conducted a 2 year treatment trial of BV (n=130), in which women were followed at regular intervals over 12 months using self-collected sampling in the same manner proposed for this trial. Loss to follow up over 12 months was less than 20% (18, 36). 

Data analysis
Data will be cleaned and entered into an Access database.  Analyses will be intention-to-treat.  Kaplan Meier methods will be used to generate survival curves for time until recurrence of BV and abnormal flora.  Recurrence rates and their 95%CI will be calculated.  Behavioural factors associated with recurrence of BV and abnormal flora will be investigated using a discrete time version of the proportional hazards regression model as proposed by Carlin and colleagues (38). Rate ratios and robust standard errors will be calculated using this methodology.
Randomisation
Sequence generation
Women who provide written informed consent will be randomly assigned using a computer-generated sequence to one of the three study arms. An independent MSHC researcher, with no other input into this trial, will generate and hold the random number sequence. Randomisation will occur in blocks of 15 to ensure that the allocation of women to the three arms occurs at a similar rate over the recruitment period.

Allocation concealment
All treatments for this trial will be identified by a unique trial number, which will tally with the randomisation schedule. Once a woman is enrolled in the study, the RA will telephone the holder of the randomisation schedule to obtain her group allocation. The sequence will be concealed until data analysis is complete.

Blinding
Participants, investigators, research staff, treating clinicians and those assessing the microbiological specimens will all be blinded as to which arm of the trial each participant is allocated. The success of blinding will be tested in the participants with a survey question.

Time Line

Time	Activity	
July-October 2007	Advertise and employ research assistant.
Educate MSHC staff about the trial.
Insert prompt on MSHC computer system
Generation of randomisation schedule
Obtain all interventions and placebos – label with unique study numbers
Assemble study kits	
November 2007 to 2009	Recruitment through MSHC 	
October to February 2010	Data entry
Data analysis
Report writing	

Potential sources of bias and how these will be handled
This trial has been rigorously designed to minimise bias. Potential sources of bias and methods to minimise them are:
·	Selection bias: use of a computer prompt, training of MSHC doctors, and availability of a research nurse to undertake all recruitment, will ensure that all women with a diagnosis of clinical BV will be offered participation in the trial; 
·	Allocation concealment will be maintained by use of a staff member with no other involvement in the trial who will generate and hold the randomisation schedule;
·	Demographic and epidemiological data for all clinic attendees is collected routinely. De-identified epidemiological data of women who decline to take part in the trial will be compared with participants to ensure that the trial participants are representative of the entire eligible population;
·	Treatment bias will be minimised through blinding of treating clinicians and pharmacists to which invention participants have been allocated;
·	Ascertainment bias: the use of a laboratory defined outcome measure, whereby the laboratory staff have no access to the clinical details or intervention group of participants; 
·	Attrition bias: scrupulous follow-up of participants and intention to treat analysis will guard against baseline imbalance in interventions groups;
·	We have calculated an adequate sample size to avoid type I and II errors.

Major anticipated confounding factors and how these will be handled
By measuring all known factors that can influence the development and recurrence of BV at baseline, and ensuring an adequate sample size, this RCT should be able to account for all known and unknown confounders.

Details of contingencies:
Participants will be counselled at recruitment regarding the potential side effects of all medications being used. This verbal information will be reinforced with written information about possible side-effect and how to manage them. All participants will be provided with a card which lists the free study telephone number to call during office hours and will connect to an investigators' mobile number after hours. Finally, participants will be informed to stop using any study medications that they feel may be causing an adverse event, if they are unable to contact a researcher to discuss. 

Details of handling of adverse events:
Serious adverse events with any of the study medications are extremely unlikely, as all medicines being tested have histories of safe usage, including Gynoflor , which is available over-the-counter in Europe. For this reason, a data monitoring and safety committee will not be formed for this study. Anticipated side effects from the antibiotics are likely to be mild and to include symptoms such as nausea, headache, heartburn, diarrhoea, vaginal discharge or a metallic taste. Side-effects of Gynoflor include local irritation and burning, and vaginal discharge.  Any serious adverse event is very unlikely; however, as stated above all participants will be given clear printed instructions on a card with 9am to 9pm daily contact details. Women will be provided with their results by study investigators and if treatment of BV is required during follow-up prompt review will be arranged with a sexual health physician at Melbourne Sexual Health Centre. All clinicians at this service are highly experienced in the management of BV. Serious adverse or unforeseen events will be managed by referral of the participant to the appropriate professional for assessment and management and will be reported to the relevant ethics committee, VMIA (Victorian Managed Insurance Authority) and the TGA.

Details of methods to ensure confidential handling and storage of records:
All participants' records will be kept in a locked cabinet at MSHC, which can only be accessed by investigators. MSHC is protected by security alarms and monitored by a security company after hours. Participants' records will be identified only by a unique code. Personal identifying details will be stored securely and kept separately from the study records, also in a locked cabinet. Only study investigators will have access to the records and codes, which will be stored indefinitely, in accordance with the Alfred Hospital requirement. All electronic records files will be subject to the same security as hard copies, in addition to being password protected, and will not be removed from MSHC. They will contain no patient identifiers.

References
1. Koumans EH, Kendrick JS. Preventing adverse sequelae of bacterial vaginosis: a public health program and research agenda. Sex Transm Dis 2001;28:292-7
2. Taha TE, Hoover DR, Dallabetta GA, et al. Bacterial vaginosis and disturbances of vaginal flora: association with increased acquisition of HIV. Aids 1998;12:1699-706
3. Hillier SL, Krohn MA, Rabe LK, Klebanoff SJ and Eschenbach DA. The normal vaginal flora, H2O2-producing lactobacilli, and bacterial vaginosis in pregnant women. Clin Infect Dis 1993;16 Suppl 4:S273-81
4. Hillier S. Normal vaginal flora. In: Holmes KK, Mardh PM, Sparling PF, et al., eds. Sexually Transmitted Diseases, 1999:191-203
5. Sobel JD. Bacterial vaginosis. Annu Rev Med 2000;51:349-56
6. Lamont RF, Morgan DJ, Wilden SD and Taylor-Robinson D. Prevalence of bacterial vaginosis in women attending one of three general practices for routine cervical cytology. Int J STD AIDS 2000;11:495-8
7. Goldenberg RL, Klebanoff MA, Nugent R, Krohn MA, Hillier S and Andrews WW. Bacterial colonization of the vagina during pregnancy in four ethnic groups. Vaginal Infections and Prematurity Study Group. Am J Obstet Gynecol 1996;174:1618-21
8. Hay PE, Lamont RF, Taylor-Robinson D, Morgan DJ, Ison C and Pearson J. Abnormal bacterial colonisation of the genital tract and subsequent preterm delivery and late miscarriage. Bmj 1994;308:295-8
9. Ralph SG, Rutherford AJ and Wilson JD. Influence of bacterial vaginosis on conception and miscarriage in the first trimester: cohort study. Bmj 1999;319:220-3
10. McGregor JA, French JI, Parker R, et al. Prevention of premature birth by screening and treatment for common genital tract infections: results of a prospective controlled evaluation. Am J Obstet Gynecol 1995;173:157-67
11. Hillier SL, Nugent RP, Eschenbach DA, et al. Association between bacterial vaginosis and preterm delivery of a low-birth-weight infant. The Vaginal Infections and Prematurity Study Group. N Engl J Med 1995;333:1737-42
12. Govender L, Hoosen AA, Moodley J, Moodley P and Sturm AW. Bacterial vaginosis and associated infections in pregnancy. Int J Gynaecol Obstet 1996;55:23-8
13. Sewankambo N, Gray RH, Wawer MJ, et al. HIV-1 infection associated with abnormal vaginal flora morphology and bacterial vaginosis. Lancet 1997;350:546-50
14. Cohen CR, Duerr A, Pruithithada N, et al. Bacterial vaginosis and HIV seroprevalence among female commercial sex workers in Chiang Mai, Thailand. Aids 1995;9:1093-7
15. Martin HL, Richardson BA, Nyange PM, et al. Vaginal lactobacilli, microbial flora, and risk of human immunodeficiency virus type 1 and sexually transmitted disease acquisition. J Infect Dis 1999;180:1863-8
16. Sexually transmitted diseases treatment guidelines 2002. Centers for Disease Control and Prevention. MMWR Recomm Rep 2002;51:1-78
17. Koumans EH, Markowitz LE and Hogan V. Indications for therapy and treatment recommendations for bacterial vaginosis in nonpregnant and pregnant women: a synthesis of data. Clin Infect Dis 2002;35:S152-72
18. Bradshaw CS, Morton AN, Hocking J, et al. High recurrence rates of bacterial vaginosis over 12 months following oral metronidazole & factors associated with recurrence. Journal of Infectious Diseases In press
19. Fredricks DN, Fiedler TL and Marrazzo JM. Molecular identification of bacteria associated with bacterial vaginosis. N Engl J Med 2005;353:1899-911
20. Fredricks DN, Marrazzo JM. Molecular methodology in determining vaginal flora in health and disease: its time has come. Curr Infect Dis Rep 2005;7:463-70
21. Vasquez A, Jakobsson T, Ahrne S, Forsum U and Molin G. Vaginal lactobacillus flora of healthy Swedish women. J Clin Microbiol 2002;40:2746-9
22. Antonio MA, Rabe LK and Hillier SL. Colonization of the rectum by Lactobacillus species and decreased risk of bacterial vaginosis. J Infect Dis 2005;192:394-8
23. McGroarty JA. Probiotic use of lactobacilli in the human female urogenital tract. FEMS Immunol Med Microbiol 1993;6:251-64
24. Klebanoff SJ, Hillier SL, Eschenbach DA and Waltersdorph AM. Control of the microbial flora of the vagina by H2O2-generating lactobacilli. J Infect Dis 1991;164:94-100
25. Hillier SL. The complexity of microbial diversity in bacterial vaginosis. N Engl J Med 2005;353:1886-7
26. Cardone A, Zarcone R, Borrelli A, Di Cunzolo A, Russo A and Tartaglia E. Utilisation of hydrogen peroxide in the treatment of recurrent bacterial vaginosis. Minerva Ginecol 2003;55:483-92
27. Wilson JD, Shann SM, Brady SK, Mammen-Tobin AG, Evans AL and Lee RA. Recurrent bacterial vaginosis: the use of maintenance acidic vaginal gel following treatment. Int J STD AIDS 2005;16:736-8
28. Holley RL, Richter HE, Varner RE, Pair L and Schwebke JR. A randomized, double-blind clinical trial of vaginal acidification versus placebo for the treatment of symptomatic bacterial vaginosis. Sex Transm Dis 2004;31:236-8
29. Parent D, Bossens M, Bayot D, et al. Therapy of bacterial vaginosis using exogenously-applied Lactobacilli acidophili and a low dose of estriol: a placebo-controlled multicentric clinical trial. Arzneimittelforschung 1996;46:68-73
30. Ozkinay E, Terek MC, Yayci M, Kaiser R, Grob P and Tuncay G. The effectiveness of live lactobacilli in combination with low dose oestriol (Gynoflor) to restore the vaginal flora after treatment of vaginal infections. Bjog 2005;112:234-40
31. Eriksson K, Carlsson B, Forsum U and Larsson PG. A double-blind treatment study of bacterial vaginosis with normal vaginal lactobacilli after an open treatment with vaginal clindamycin ovules. Acta Derm Venereol 2005;85:42-6
32. Hallen A, Jarstrand C and Pahlson C. Treatment of bacterial vaginosis with lactobacilli. Sex Transm Dis 1992;19:146-8
33. Shalev E, Battino S, Weiner E, Colodner R and Keness Y. Ingestion of yogurt containing Lactobacillus acidophilus compared with pasteurized yogurt as prophylaxis for recurrent candidal vaginitis and bacterial vaginosis. Arch Fam Med 1996;5:593-6
34. Neri A, Sabah G and Samra Z. Bacterial vaginosis in pregnancy treated with yoghurt. Acta Obstet Gynecol Scand 1993;72:17-9
35. Shoubnikova M, Hellberg D, Nilsson S and Mardh PA. Contraceptive use in women with bacterial vaginosis. Contraception 1997;55:355-8
36. Bradshaw CS, Morton AN, Garland SM, Morris MB, Moss LM and Fairley CK. Higher-risk behavioral practices associated with bacterial vaginosis compared with vaginal candidiasis. Obstet Gynecol 2005;106:105-14
37. Calzolari E, Masciangelo R, Milite V and Verteramo R. Bacterial vaginosis and contraceptive methods. Int J Gynaecol Obstet 2000;70:341-6
38. Carlin, J., R. Wolfe, et al. (1999). " Tutorial in Biostatistics. Analysis of binary outcomes in longitudinal studies using weighted estimating equations and discrete-time survival methods: prevalence and incidence of smoking in an adolescent cohort." Statistics in Medicine 18: 2655-2670
